# An Easy-to-Implement Protocol for Preparing Postnatal Ventral Mesencephalic Cultures

**DOI:** 10.3389/fncel.2018.00044

**Published:** 2018-03-02

**Authors:** Janin Lautenschläger, Eugene V. Mosharov, Ellen Kanter, David Sulzer, Gabriele S. Kaminski Schierle

**Affiliations:** ^1^Department of Chemical Engineering and Biotechnology, University of Cambridge, Cambridge, United Kingdom; ^2^Department of Psychiatry, College of Physicians and Surgeons, Columbia University, New York, NY, United States; ^3^Division of Molecular Therapeutics, New York State Psychiatric Institute, New York, NY, United States

**Keywords:** ventral mesencephalic neurons, dopaminergic neurons, postnatal neuron cultures, substantia nigra, ventral tegmental area, postnatal neurons, protocol, Parkinson’s disease

## Abstract

Postnatally derived cultures of ventral mesencephalic neurons offer several crucial advantages over embryonic ventral mesencephalic cultures, including a higher content of TH-positive cells and the ability to derive cells from the substantia nigra, which contains the neurons most vulnerable to Parkinson’s disease. On the other hand, these cultures are more challenging to produce consistently. Here, we provide an easy-to-implement protocol for culturing postnatal ventral mesencephalic cells from the substantia nigra (SN) and the ventral tegmental area using commercially available media, dishes, and general lab equipment, avoiding extensive material and equipment purchases. The protocol can be completed in about 5 h and provides ventral midbrain neuron cultures on cortex glia feeder layers in three weeks’ time. The protocol uses an optimized protease digestion, tissue storage in Hibernate A during dissection and purification of neurons on an OptiPrep density gradient.

## Introduction

When neuronal cell lines are not sufficient to study the aspects of neuron physiology and pathophysiology, primary neuronal cultures can often be used instead. Primary neuronal cultures can be derived either from embryonic or postnatal tissue and both have their advantages and disadvantages depending on the scientific question at hand. Embryonic ventral midbrain (VM) neuronal cultures are easier to prepare, however they have the drawback of only containing 1–2% of Tyrosine hydroxylase (TH) -positive cells, limiting quantitative and single cell analyses. Conversely, primary postnatal cultures are more difficult to derive, but contain a substantial amount of TH-positive cells. Furthermore, neurons from either the substantia nigra (SN) or the ventral tegmental area (VTA) can be isolated separately, as shown and characterized in previous papers (Masuko et al., 1992; Burke et al., 1998; Mosharov et al., 2009), enabling studies comparing cells, which are either more vulnerable (SN) or less vulnerable (VTA) in patients suffering from Parkinson’s disease (PD).

Postnatal rodent midbrain cultures were introduced 25 years ago (Rayport et al., 1992) and have been extensively employed since. It was observed that cell survival drops significantly if the pups are older than 2 days postpartum, limiting the time at which neurons can be obtained for culturing. Nevertheless, we and others have demonstrated several important physiological characteristics of cultured ventral mesencephalic neurons, including (1) stimulation-dependent dopamine exocytosis (Pothos et al., 2000; Staal et al., 2004; Mosharov et al., 2009), (2) the presence of ‘adult’ plasma membrane channels (Rayport et al., 1992; Sulzer et al., 1998; Dryanovski et al., 2013), and (3) morphological differences between SN and VTA neurons (Dryanovski et al., 2013; Pacelli et al., 2015). Previously, we demonstrated that whereas total VM (SN+VTA) cultures contain ∼50% of TH-positive cells that are also calbindin-positive, this proportion is ∼25% in SN and ∼85% in VTA cultures, mirroring the *in situ* distribution of calbindin positive neurons (Thompson, 2005).

One of the drawbacks of both early (Masuko et al., 1992; Rayport et al., 1992; Cardozo, 1993) and newer protocols (Smeyne and Smeyne, 2002; Fasano et al., 2008; Frank et al., 2008) for preparing postnatal ventral mesencephalic cultures is that in general they are more elaborate in terms of media and reagent set up compared to protocols for embryonic cultures. A simple PubMed query shows that embryonic dopaminergic neuron cultures are preferentially used (1819 publications) and postnatal dopaminergic neuron protocols have not become a general tool (484 publications). This paper aims to provide an easy-to-implement protocol for the culture of postnatal ventral mesencephalic neurons by using standard media for adult neuronal cultures, minimizing reagent setup and not having the need of oxygenation, thus encouraging the transition from embryonic to postnatal ventral mesencephalic cultures. Applications for this protocol mainly lie in the field of PD, for example enabling the study of differential toxicity of MPP^+^ (1-methyl-4-phenylpyridinium) in SN versus VTA neurons or the effects of cytosolic dopamine, calcium and alpha-synuclein levels. However, this protocol is also interesting for the understanding of physiological properties of ventral mesencephalic neurons such as the mechanism of dopamine release (Staal et al., 2004; Mosharov et al., 2009; Dryanovski et al., 2013; Pacelli et al., 2015; Lieberman et al., 2017).

## Experimental Design

**FIGURE 1:**
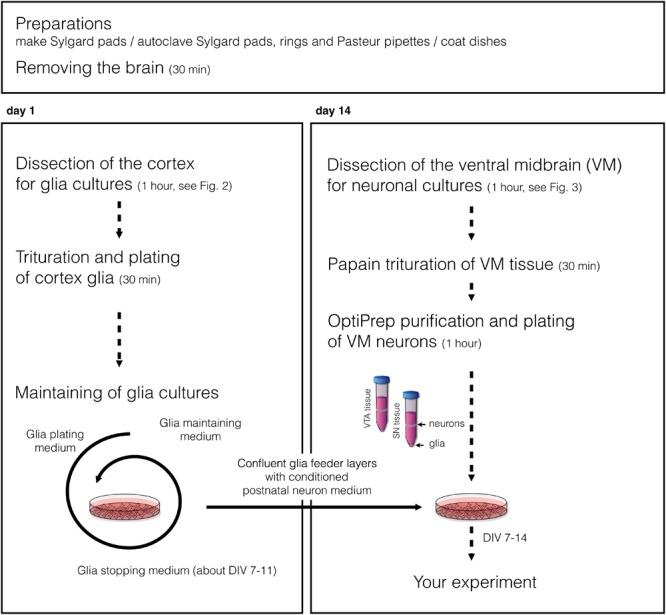
Flow chart diagram of experimental design.

The protocol describes a culture system where ventral mesencephalic cells are maintained on a glia feeder layer. Thus, cortex glia cultures need to be obtained first (day 1), while ventral mesencephalic neurons are obtained in a second dissection and plated onto by then confluent glia feeder layers (day 14). Cultures can be obtained from rat as well as mouse brain (P0-P2). **Figure 1** presents the flow chart of the protocol.

### Glia Feeder Layers

**FIGURE 2:**
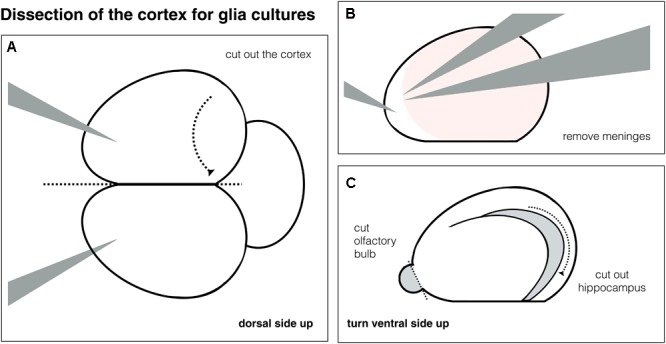
Schematic of cortex dissection. **(A)** Whole brain and dissection of one cortex. **(B)** Removal of meninges from the cortex. **(C)** Removal of olfactory bulb and the hippocampus.

First, the brain needs to be removed from the skull, which takes about 30 min, with less than 2 min per brain (12–15 rat pups per litter). Then the cortex tissue for glia feeder layers is dissected (1 h, **Figure 2**). The tissue can be triturated directly without enzymatic digestion, cells are plated on poly-L-lysine coated glass-bottom dishes (30 min). Critical steps during maintaining glia cultures include a rough wash the day after plating to remove non-attached cells and a mitotic inhibition after reaching confluency.

### Ventral Mesencephalic Cultures

**FIGURE 3:**
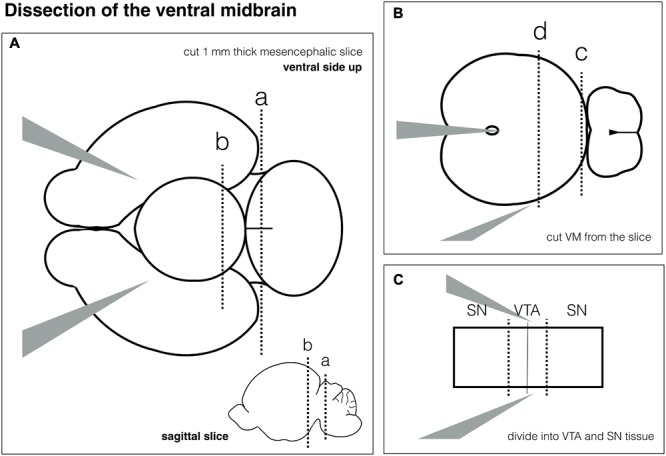
Schematic for ventral midbrain (VM) dissection. **(A)** Dissection of mesencephalic slice from the whole brain. **(B)** Dissection of the VM. **(C)** Separation of Substantia nigra (SN) and ventral tegmental area (VTA).

One or 2 days before the dissection of VM neurons, confluent glia feeder layers receive postnatal neuron medium to condition the media before the neurons are plated on top. The dissection of the VM takes about 1 h, with less than 5 min per brain (**Figure 3**). The tissue is enzymatically digested using papain and triturated afterward to obtain a single cell suspension (30 min). The cell suspension is centrifuged on an OptiPrep density gradient to purify neurons and to remove cell debris. Neurons are resuspended and plated in MatTek dishes with glia feeder layers and postnatal neuronal medium. GDNF (glial cell derived neurotrophic factor) is added as a growth factor (altogether 1 h). The same brains can be used to obtain new glia cultures from cortex if a second VM dissection is planned.

## Materials and Equipment

### Materials and Reagents

All materials and reagents needed for the coating process, the dissection itself and for the neuron and glia media are given in **Table 1**.

**Table 1:** Overview of materials and reagents.

	Product code	Price in USD
**Coating process**		
MatTek dishes	MatTek Cor., P35G-1.5-14-C, 200 units	335
Poly-L-lysine	Sigma–Aldrich^®^, P4707-50ML	85
Sterile water	GElifesciences, SH30221.17, 500 mL	15
**Dissection**		
Minisart filter 0.2 μm	Sigma–Aldrich^®^, 16534K, 50 units	161
10 mL Syringe	BD Plastipak, 302188, 100 units	9
Sylgard^®^ 184 kit	Dow Corning^®^, 184S1.1, 1.1 KG	122
Sterile PBS	Gibco, 14190250, 10x 500 mL	174
45% Glucose solution	Sigma–Aldrich^®^, G8769-100ML	30
Hibernate^®^ A minus Calcium	BrainBits UK, HA-Ca, 500 mL	110
Papain suspension	Worthington Bio. Cor., LS003126, 100 mg	70
DNase I	Sigma–Aldrich^®^, DN25-100MG	75
OptiPrep Density Gradient	Sigma–Aldrich^®^, D1556-250ML	257
Pasteur pipets 230 mm	Fisher Scientific, 11566963, 1000 units	52
Slide rings 5 mm × 18 mm 12/CT	Fisher Scientific, NC0639576, 12 units	50
**Neuron media**		
Neurobasal-A medium	Life Technologies, 10888022, 500 mL	65
B-27^®^ Supplement 50x	Life Technologies, 17504044, 10 mL	90
GlutaMAX^TM^ Supplement 100x	Life Technologies, 35050038, 100 mL	30
Gentamycin 10 mg/mL	Life Technologies, 15710049, 50 mL	137
GDNF	Merck Millipore, GF030, 10 μg	359
**Glia media**		
DMEM – high glucose	Sigma–Aldrich^®^, D6546-500ML	30
FBS	Life Technologies, 10270106, 500 mL	61
Antibiotics	Gibco, 15240062, 100 mL	36
Cytarabine	Abcam, ab141924, 100 mg	39

### Equipment

Standard cell culture lab equipment, including:

• Laminar Flow Cabinet, CO_2_ cell incubator, inverted microscope.
• Hemocytometer, 0.4% Trypan Blue stain.
• Dissection stereomicroscope, Petri dishes, a flat cool pack.
• Swinging bucket centrifuge with 15 mL tube inlets.

### Dissection Instruments

• Lister Scissors, serrated 14 cm (Fine Science Tools, cat no. 14131-14), $100.75.
• Tissue Forceps, 1x2 Teeth 15.5 cm (Fine Science Tools, cat no. 11021-15), $27.00.
• Alternatively, for mice: 1x2 Teeth 12 cm (Fine Science Tools, cat no. 11021-12), $22.00.
• Standard Pattern Forceps, curved 12 cm (Fine Science Tools, cat no. 11001-12), $24.00.
• Spring Scissors, Angl. 10 mm (Fine Science Tools, cat no. 15006-09), $308.00.
• Spatula (VWR, cat no. BOCH3340), $8.10.
• 2x Dumont #5 Forceps (Fine Science Tools, cat no. 11253-20), 2x $40.50.
• 1x Dumont # 5A Forceps (Fine Science Tools, cat no. 11253-21), $38.25.
• No. 3 Scalpel handle (Swann-Morton^®^, No 3G S/S), $3.73.
• Blades No. 11 (Swann-Morton^®^, 0303100), $15.20.

NOTE! Instruments have to be cleaned and sterilized on a regular basis to preserve functionality and avoid contamination.

## Stock Solution and Media Setup

### Cytarabine 100x Stock Solution at 10 mM

• Dissolve 100 mg cytarabine with 41.115 mL sterile water (cytarabine is a chemotherapeutic agent, use chemical hood during dissolving procedure, see MSDS for further information).

• Filter sterilize and store in aliquots of 5 mL at -20°C.

### DNase 100x Stock Solution

• Dissolve 100 mg DNase in 2 mL of sterile water.
• Filter sterilize and store as 20 μL aliquots at -20°C.

### GDNF 20 μg/mL Stock Solution

• Dissolve 10 μg GDNF in 500 μL sterile water, store as 10 μL aliquots at -20°C.
• Dilute 1:2000 in Postnatal neuron medium for a final concentration of 10 ng/mL.

### Glia Plating Medium – DMEM With 10% FBS

• Add 5 mL of fetal bovine serum (FBS) to 45 mL of DMEM high glucose medium.

### Glia Stopping Medium – DMEM With 5% FBS and 1:100 Cytarabine

• Add 2.5 mL of FBS and 500 μL of the cytarabine stock solution to 47 mL of DMEM high glucose medium.

### Glia Maintaining Medium – DMEM With 5% FBS

• Add 2.5 mL of FBS to 47.5 mL of DMEM high glucose medium.

### OptiPrep Gradient 6.2%

^∗^OptiPrep^TM^ is commonly used to separate cells on a density gradient. In the same way it is able to separate intact cells from cell debris which is critical to obtain healthy cultures.

• Add 930 μL OptiPrep to 8 mL Hibernate A^®^ minus Calcium and store at 4°C, protect from light.

### Papain Dissociation Solution – 20 Units/mL

• Add appropriate volume of the papain suspension to 3 mL Hibernate A^®^ minus Calcium to achieve a concentration of 20 units/mL.

NOTE! The amount of papain is batch specific and has to be calculated from the activity per mg protein (u/mgP) and the concentration of protein per mL (mgP/mL).

• Activate in the incubator at 37°C for 30 min, then filter sterilize into a new Falcon tube.

CRITICAL STEP! The papain solution has to be made up freshly for each dissection. The solution is cloudy but should get clear after activation – see Troubleshooting point – Papain solution.

### PBS Dissection Buffer

• Add 650 μL of 45% glucose solution to 49.35 mL sterile PBS without CaCl_2_ and MgCl_2_.
• Solution needs to be cooled to 4°C.
• You will need 1x 50 mL Falcon tube per dissection, some spare solution for the glia dissociation and 2x half-filled 50 mL Falcon tubes if you need to transport pup heads.

### Postnatal Neuron Medium – Neurobasal A With 2% B27, 0.5 mM GlutaMAX and 10 μg/mL Gentamycin

• Add 200 μL B27, 25 μL GlutaMAX, and 10 μL Gentamycin to 9.8 mL of Neurobasal A medium.

CRITICAL STEP! Postnatal neuron medium has to be added to the dishes with glia cultures 1 or 2 days before neurons are plated so that media can be conditioned.

## Procedure

### Preparations

^∗^The Sylgard is used to make flat pads which function as a bumper when cutting the brain tissue during dissection. Sylgard pads, rings and Pasteur pipets need to be autoclaved before usage.

• Mix 1 g curing agent and 10 g elastomer, pour in a 12 well plate so that the liquid level is about 1–2 mm in height. Leave at room temperature for 2 days until solid.
• Wrap single pads in aluminum foil, autoclave and keep until dissection.
• Wrap single slide rings in aluminum foil, autoclave and keep until dissection.
• Put Pasteur pipets in steel canister and autoclave.

^∗^For appropriate cell attachment, glass-bottom dishes (MatTek dishes, glass culture dishes from Electron Microscopy Science (70760), or custom made glass bottom dishes) have to be coated, Poly-L-lysine has been proven to be sufficient. The coating can be done the day before, if done longer in advance feeder layers may take longer to achieve confluency.

• Put a drop of about 200 μL Poly-L-lysine solution onto the glass surface.
• Leave for 1 h, wash 3x times with sterile water and let dry in the Laminar Flow Cabinet.

^∗^Meanwhile, you can prepare the Postnatal neuron medium, which has to be added to the dishes with the pre-established glia feeder layers. Glia plating media can be prepared in advance as well. Please refer to STOCK SOLUTIONS AND MEDIA for recipes.

### Removing the Brain – 30 min

^∗^Please note that all animal experiments underlie the respective national authorities. Animals used were bred and supplied by Charles River UK, Ltd., Scientific, Breeding and Supplying Establishment, registered under Animals (Scientific Procedures) Act 1986, and AAALAC International accredited. This study was carried out in accordance with the recommendations of the Animals (Scientific Procedures) Act 1986 provided by the UK Home Office. The protocol was approved by the NACWO and University of Cambridge Ethics Board.

• Decapitate rat pups (P0-P2) using the Listor scissors. The heads can be used fresh, or stored in PBS dissection buffer for up to 2 h.
• Put heads onto a 9–10 cm Petri dish lid, hold the heads using the teeth forceps grabbing at the level of the eyes.
• Remove the skin from the skull using the curved forceps grabbing a large skin fold and pulling lightly cranial, then caudal.
• Cut round the skull with the spring scissors. First cut the half facing away from you, pointing the scissors away from you. Then do the same for the side facing toward you, scissors pointing toward you. Make sure to cut a full circle including the sutura sagittalis.
• The skullcap can now be removed easily in one piece using the fine forceps No. 5.
• Remove the brain using the small spoon of the spatula.

TIP! Set up a 9–10 cm Petri dish under the dissection stereomicroscope, stick the Sylgard pad with the concave side facing up onto the bottom and fill with 50 mL cooled PBS dissection buffer prior to removing the brain. Make sure not to touch the liquid with unsterile parts. An ice pad underneath helps to cool the solution during the dissection.

### Dissection of the Cortex – 1 h

^∗^The cortex tissue is needed to obtain glia feeder layers for the neuronal cultures. For cortex dissection see **Figure 2** (The cortex can also be obtained from brains used for VM dissection before).

• Put the brain onto the Sylgard pad, with the dorsal side facing up. Make sure the brains are fully submerged in PBS.
• Cut the cortex by inserting the scalpel between the two hemispheres, then carefully divide the cortex from the diencephalon – see **Figure 2A**.
• Remove the meninges using the forceps 5A and 5, one holding the tissue, one gently pulling – see **Figure 2B**, check dorsal and ventral side are free of meninges.
• Remove the olfactory bulb and the hippocampus – **Figure 2C**.
• Store tissue in PBS dissection buffer, put on ice. Pause point – tissue can be kept for up to 2–3 h before trituration (if both VM and glia cultures are done at the same day, VM tissue can be obtained and triturated first, while cortex tissue is maintained on ice).

### Trituration of Glia Cells – 30 min

^∗^The glia cells can be triturated with a simpler approach than for neurons, as neuronal viability is not intended at this point. From one half of the pup cortex, you can obtain about 2 million cells. Cells reach confluence after about 7–11 days in vitro.

• Put 5 mL of the PBS dissection buffer in a 50 mL Falcon tube and transfer the cortex tissue into the Falcon tube.
• Triturate 2x 15 times with a FBS-coated 1000 μL pipet tip.
• Mix 10 μL of cell suspension with 10 μL Trypan blue stain and load 9 μL onto the Hemocytometer, count unstained cells.
• Let tissue chunks sediment, take 5 mL of single cell suspension in a new Falcon tube.
• Centrifuge at 750 *g* for 5 min and resuspend cell pellet in 1 mL of Glia plating media.
• Plate 650,000 cells per MatTek dish.

TIP! You can already prepare the dishes with Glia plating medium (10% FBS in DMEM), then putting the cells in the middle on the coverslip. Let them sediment briefly for 1 or 2 min, then swirl cells gently around and put dishes into the incubator.

### Maintenance of Glia Cultures

^∗^There are some critical steps in the maintenance of the glia cells to get high quality monolayers. Cells must be washed rigorously and cell division has to be stopped at the right time point. The media changes can be done using media at 4°C, as this helps to retain cultures free from neurons.

• The day after dissection glia cultures must be washed to remove non-attached cells.

TIP! Use a 5 mL stripette with a pipetboy for this washing step. Take up the old medium and rigorously pipette the media directly onto the cells on the cover slip, repeat five times. If you have done this properly, only a few attached cells should be left on the coverslip now.

• Remove the old medium and add 2 mL of fresh Glia plating medium.
• Glia medium needs to be changed twice a week using Glia plating medium (10% FBS in DMEM).

TIP! Watch your cells carefully before each media change. With confluency single glia cells take an angular shape within the monolayer (see **Figure 6**). If feeder layers are stopped too late, microglia, small round cells with high phase contrast, might enrich on top of the monolayer.

• Upon reaching confluence (normally between DIV 7 and 11) cell division has to be halted by changing media to Glia stopping medium (5% FBS in DMEM plus cytarabine).
• With the next regular media change, wash cells carefully and put Glia maintaining medium.

TIP! Before removing glia stopping medium, use a 1000 μL pipet tip to swirl medium around, repeat five times. This will help to remove microglia, which are loose after the treatment with Glia stopping medium. Discard media according to national waste disposal guidelines as it contains cytarabine.

• The twice weekly media change should be done with Glia maintaining medium (5% FBS in DMEM) from now on.

• Please also see Troubleshooting point – Maintenance of glia cultures.

### Dissection of the Ventral Midbrain (VM) for Neuronal Cultures – 1 h, See **Figure 3**

^∗^This section describes how to dissect the ventral midbrain from postnatal rodent brain. The VM is isolated using a slice cutting approach and can be separated into VTA and SN. **Figure 3** gives a schematic overview of individual steps.

• Put the brain onto the Sylgard pad with the ventral side facing up to see the midbrain flexure.
• Hold the brain by inserting the fine forceps No. 5A into both hemispheres.
• Gently remove the meninges over the hindbrain using the fine forceps No. 5.
• Make a vertical cut with the scalpel at the level of the hindbrain. see **Figure 3A**, dotted line a.
• Then make another vertical cut cranial to the midbrain flexure – see **Figure 3A**, dotted line b.
• You will have an approximately 1 mm thick coronal brain slice of the mesencephalon. The IV ventricle should be visible on the caudal side of the slice.
• Put the fine forceps No. 5A into the ventricle to hold the slice – **Figure 3B**.
• Make a vertical cut between midbrain and hindbrain – see **Figure 3B**, dotted line c.
• Then make another cut half-way between the 1^st^ cut and the ventricle – see **Figure 3B**, dotted line d.

CRITICAL STEP! Sometimes there can be some meninges left. Make sure to remove them.

• Divide the VM into SN and VTA by cutting 1/3, 1/3, 1/3. See dotted lines in **Figure 3C**.
• Put tissue chunks in a 1.5 mL Eppendorf tubes containing cooled Hibernate A^®^ minus Calcium, using a transfer pipette, put back on ice.
• Repeat all steps with the next brain.
• Pause point – tissue can be kept for about an hour in Hibernate A^®^ minus Calcium before papain digestion is started.

### Papain Digestion of VM Tissue – 30 min

^∗^The dissociation of VM tissue is done using a mild papain digestion. For dissociation, a standard 1000 μL pipet tip coated with FBS is used. The papain can be prepared before starting the dissection. Filtered solution can be kept at 37°C for up to 3 h, otherwise store at 4°C.

• Remove Hibernate A^®^ minus Calcium from the tube with the tissue chunks.
• Add 1 mL of the activated and filtered papain solution.
• Incubate for 10 min in the incubator at 37°C.
• In the meantime, prepare the washing solution – add 20 μl of the DNase stock solution to 2 mL Hibernate A^®^ minus Calcium, prepare twice if you do SN and VTA separately.
• After 10 min take your cells from the incubator and remove the papain solution, rinse 4x with 500 μL of washing solution each time.
• Add 1 mL of Hibernate A^®^ minus Calcium.
• Triturate with a FBS-coated 1000 μL pipet tip.
• About 30–40 trituration steps in total should be sufficient to reach a single cell suspension, let tissue chunks settle after every 10 steps.

TIP! Use a FBS coated pipet tip. For this, pipette up and down several times in a small volume of FBS before triturating papain-digested tissue chunks.

### OptiPrep Purification and Plating of VM Cells – 1 h

^∗^The OptiPrep purification is a critical step to obtain healthy neuron cultures. Neurons can survive without this step but cell debris can interfere with neuronal sprouting and development.

• Put 1 mL of cell suspension in a 15 mL Falcon tube and add 3 mL of Hibernate A^®^ minus Calcium.
• Use the long Pasteur pipet to add a sublayer of 1–1.5 mL of the 6.2% OptiPrep solution.
  CRITICAL STEP! Make sure to obtain two separate layers. Take up the OptiPrep solution with a long Pasteur pipet, carefully go to the bottom of the Falcon tube and slowly release the OptiPrep solution.
• Centrifuge for 15 min at 500 *g*, do not use the brake to avoid disturbing the layers.
• After centrifugation remove about 3 mL from the top containing cell debris.
• Collect cells by harvesting the turbid band just in between the two layers, put these 1–2 mL in a new 15 mL Falcon tube.
• Add the same volume of Hibernate A^®^ minus Calcium to your cells and centrifuge for 3 min at 500 *g*, no brake.
• Re-suspend your pellet in 1 mL Hibernate A^®^ minus Calcium.
• Count your cells by loading 9 μL of the cell suspension onto the Hemocytometer.
• According to the experimental setup plate 50,000 to 100,000 cells in the middle of each MatTek dish with the pre-established glia feeder layers and the pre-conditioned Postnatal neuron medium, let cells settle for 10–15 min.
• Add 1 μL of GDNF stock solution to each dish with 2 mL media (10 ng/mL) (Burke et al., 1998).
• The next day add 2 μL of the cytarabine stock solution to 2 mL media to avoid glia growth.

TIP! Slide rings can be used to keep the cells in the middle of the dish. Here, cell numbers should be reduced. Rings can be reused, wash with plenty of water, spray with ethanol and autoclave again.

• Put one of the autoclaved slide rings in the middle of each MatTek dish with the pre-established glia feeder layers and the pre-conditioned Postnatal neuron medium.
• Plate your cells in the middle of the slide ring.
• Remove the slide rings the next day. Use forceps covered by sterile non-filter pipet tips to avoid contamination.
  The cells can be used at DIV 7-14, but also maintenance up to DIV 48 is possible.

TIP! Try to keep your cells without media change, they are healthier with conditioned media. If you must change media, keep half of the old media and add half of fresh preconditioned media.

## Results

**FIGURE 4:**
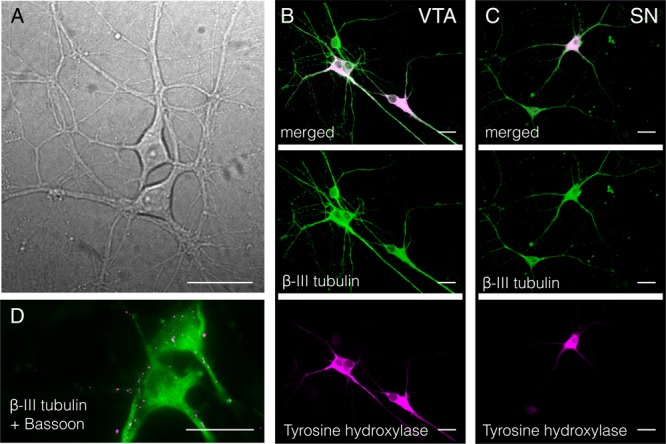
Rat ventral midbrain cultures. **(A)** Brightfield image of live cultures. **(B,C)** β-III tubulin and tyrosine hydroxylase co-staining for VTA and SN cultures. **(D)** β-III tubulin and bassoon co-staining showing the development of synapses. Scale bars represent 30 μm.

**FIGURE 5:**
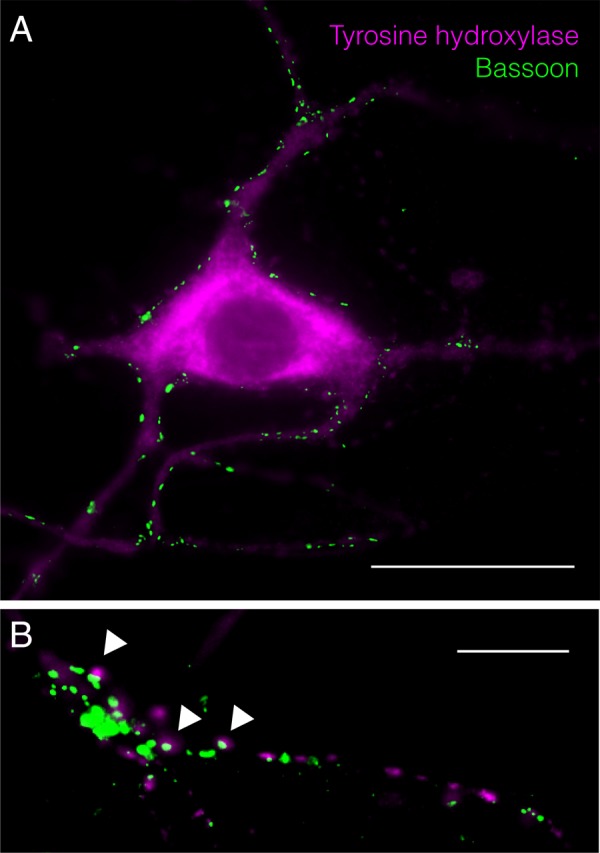
Tyrosine hydroxylase and bassoon co-staining for VM cultures. **(A)** Synaptic inputs on TH-positive cell. Scale bar represent 30 μm. **(B)** TH-positive axon terminals with bassoon co-labeling. Scale bar represent 10 μm.

Postnatal ventral mesencephalic cultures obtained with this protocol are shown in **Figure 4A**. The cultures show 40.86 ± 6.24% and 34.10 ± 2.40% of TH-positive cells for VTA and SN cultures respectively, as determined by beta-III tubulin (ab7751, Abcam, Cambridge, United Kingdom)/tyrosine hydroxylase (ab41528, Abcam) co-staining (**Figures 4B,C**). The presence of synaptic connections is shown by co-labeling for the presynaptic marker bassoon (ab82958, Abcam) and beta-III tubulin (ab7751, Abcam, Cambridge, United Kingdom) (**Figure 4D**). TH-positive ventral mesencephalic cells are highly connected within the neuronal network, showing multiple synaptic inputs (**Figure 5A**) as well as TH-positive axon terminals co-labeled with bassoon (**Figure 5B**, arrowheads). Further experiments, including electrophysiology, are required to fully characterize these cultures.

## Conclusion

Cultures of postnatal ventral mesencephalic neurons have a wide range of applications, in the study of PD as well as in studying physiologic properties of neuronal subtypes. The culture protocol we present here uses standard media for postnatal neuron cultures and avoids extensive media and equipment setup, thus giving an easy start up protocol for everyone aiming to implement postnatal ventral mesencephalic cultures.

## Troubleshooting

### Troubleshooting Point – Papain Solution

The papain solution should be clear after activation at 37°C. If this is not the case, the papain is not adequately dissolved and crystals can rupture cells membranes. The protocol from Worthington Biochemical Corporation recommends adding EDTA, 2-mercaptoethanol and cysteine HCl to dissolve papain (see papain datasheet for details). Please note that in this case the solution needs to be pH adjusted afterward. In general, the papain suspension should dissolve in Hibernate A^®^ minus Calcium, if this is not the case the medium might have lost its oxide reductase activity.

### Troubleshooting Point – Maintenance of Glia Cultures

**FIGURE 6:**
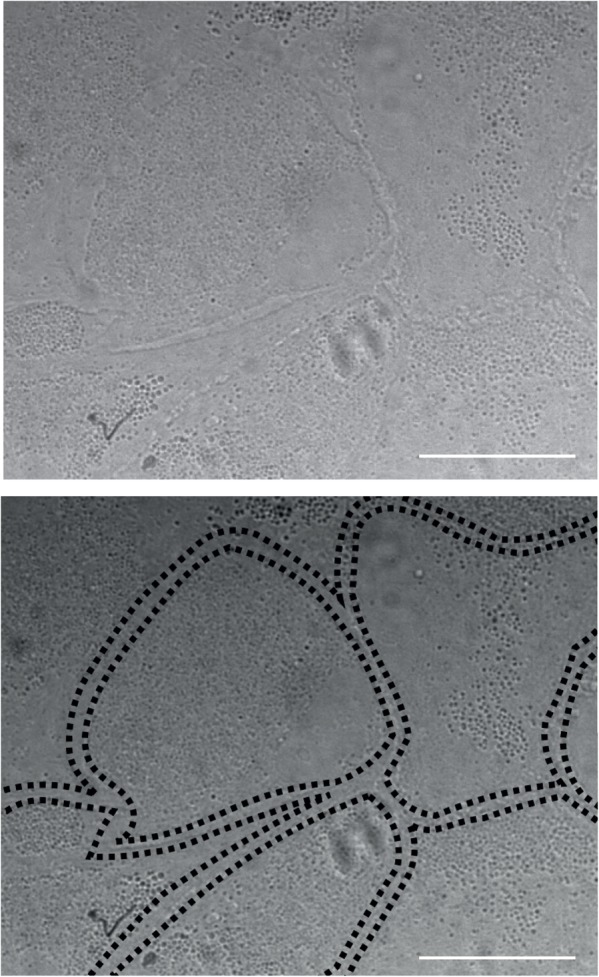
Maintenance of glia feeder layers. Optimal glia feeder layer with closely packed cells in an angular shape. Scale bars represent 30 μm.

It is critical to stop glia cells from dividing in order to achieve good feeder layers, which provide optimal support for neuronal cultures afterward. For this, check your glia cultures regularly. In general, they need Glia stopping medium between DIV 7-11, however, timing may vary depending on the seeding density and quality of the coating. Optimal glia feeder layers show an angular shape of single cells when they are closely packed (**Figure 6**). If cell division is stopped too early, glia cells do not form a continuous monolayer, however if cell division is stopped too late, microglia settle on top of the feeder layer and can have neurotoxic effects. Microglia may be removed with the media change after stopping with cytarabine using rough washing.

The glia culture protocol used here was adapted from (Pébay et al., 2001; Rouach et al., 2006), however, other protocols using papain digestion of cortical tissue or passaging of glia in cell culture flasks can give similar outcomes and can substituted if favored.

### Troubleshooting Point – Neurons Don’t Survive

1. When implementing cultures, it is good to start with mixed VM cultures, as this will give you an idea if the protocol works before going to the more vulnerable SN cultures. If you make mixed VM cultures, cut tissue into small chunks to ensure appropriate digestion during papain incubation.
2. In general, cultures should be handled with care. Avoid shaking and extensive times out of the incubator. Do not place dishes on cold steel surfaces, but rather use a heating pad or pre-warmed plastic support underneath.
3. Make sure to have good feeder layers. If glia cells are too sparse, the neuronal cultures are not sufficiently supported, on the other hand if the glia feeder layers are too dense, the postnatal neuron medium is exhausted too quickly and you need to change media.
4. The B27 is a critical supplement in the neuronal medium, however, it can vary from batch to batch. It is therefore, good practice to try the new batch in comparison with the old one before running out.
5. Additionally acetylcysteine, desferoxamine, and kynurenic acid have been shown to have beneficial effect on postnatal VM neuron cultures and can be tried to improve culture conditions (Mena et al., 1997; Sulzer et al., 2000; Petersen et al., 2001).

## Author Contributions

JL carried out the experiments. JL wrote the manuscript with support from EM, EK, DS, and GKS. DS and GKS helped supervise the project. All authors were involved in the conception of the work, discussed the results, approved the final manuscript, and agreed to be accountable for all aspects of the work.

## Conflict of Interest Statement

The authors declare that the research was conducted in the absence of any commercial or financial relationships that could be construed as a potential conflict of interest.
